# “Talking to Someone Who Gets It…” Experiences of Surgical Site Teams in Implementing a Peer Support Programme for Surgeons After Adverse Events

**DOI:** 10.3390/healthcare14091220

**Published:** 2026-05-01

**Authors:** Gemma Scrimgeour, Maddy Greville-Harris, Agata Wezyk, Kevin Thomas, Olivia Donnelly, Betul Tatar, Emma King, Ralph Tomlinson, Sheena MacSween, Kevin Turner

**Affiliations:** 1 Department of Urology, Salisbury District Hospital, Salisbury NHS Foundation Trust, Odstock Road, Wiltshire SP2 8BJ, UK; 2 Surgeon Wellbeing Research Team, School of Psychology, Poole House, Bournemouth University, Fern Barrow, Poole BH12 5BB, UK; 3 Psychology Department, University of Exeter, Washington Singer Building, Perry Road, Exeter EX4 4QG, UK; 4 Staff Psychology Team, North Bristol NHS Trust, Bristol BS10 5NB, UK; 5 Otorhinolaryngology, Head and Neck Surgery, University Hospitals Dorset, Castle Lane East, Bournemouth BH7 7DW, UK; 6 Research and Quality Improvement, Royal College of Surgeons of England, 38-43 Lincoln’s Inn Fields, London WC2A 3PE, UK; 7 Department of Urology, University Hospitals Dorset, Castle Lane East, Bournemouth BH7 7DW, UK

**Keywords:** surgeon wellbeing, adverse events, peer support, reflexive thematic analysis

## Abstract

**Highlights:**

**What are the main findings?**

- Perceptions of a multi-site peer support initiative for surgeons following adverse events (SUPPORT) were explored through in-depth interviews with site team members. Participants highlighted the perceived usefulness of the initiative, as well as the cultural and systemic barriers to implementation.
- Four key themes were identified, underlining the perceived: (1) value of SUPPORT, (2) role of SUPPORT in challenging cultural barriers around support-seeking, (3) implementational challenges such as limited protected time and difficulty identifying affected surgeons, and: (4) importance of institutional and leadership backing.

**What are the implications of the main findings?**

- Despite organisational and practical barriers to implementation, the peer support initiative was seen as a powerful tool to normalise support-seeking and ‘human’ responses to adverse events.
- Findings highlight the perceived importance of interventions that address cultural barriers for surgeons and promote acceptance of support-seeking following adverse events. Such initiatives appear to challenge surgical norms around infallibility, stigma and silence following mistakes in surgery.

**Abstract:**

**Background/Objectives**: Surgeon health and psychological functioning are profoundly impacted by adverse events in surgery. This inevitably affects workforce performance and patient safety. Despite this, routine wellbeing support for surgeons following such incidents is not routinely offered or utilised. The SUPPORT (SUrgeon Peer-led Post-incident Response Teams) Quality Improvement collaborative was developed to address this gap, supporting 12 hospital Trusts/Groups across the UK and Republic of Ireland to implement a peer support programme for surgeons. This qualitative study explored surgical site team members’ experiences of employing SUPPORT in their hospitals, focusing on the barriers and facilitators encountered in this process. **Methods**: Semi-structured interviews were conducted with 16 surgical site team members enrolled in SUPPORT (13 surgeons, 2 psychologists and 1 anaesthetist). Interviews were recorded, transcribed, and analysed using reflexive thematic analysis to identify key themes/subthemes within the data. **Results**: While there was a strong collective belief in the importance of SUPPORT, participants highlighted a disconnect between conceptual endorsement and the practical, institutional, and cultural challenges of implementation. SUPPORT was seen as an important tool in the context of adverse events, as well as a way to challenge the cultural stigma around showing vulnerability, admitting mistakes, and seeking support. **Conclusions**: This study explored important barriers and facilitators to implementing a peer support programme for surgeons, highlighting key considerations for surgeon-specific interventions in the aftermath of adverse events.

## 1. Introduction

Adverse events in surgery (AEs) are almost inevitable in any surgeon’s career [1,2]. Indeed, 80% of surgeons in a USA sample recalled at least one AE within a 12-month period [3]. While definitions of AEs tend rightly to focus on the harm inflicted upon patients, surgeons are significantly affected too; AEs lead to increased anxiety, low mood, sleep disturbance, and increased alcohol consumption [4,5]. One-third of surgeons in a UK sample (*n* = 445) reported clinically significant post-traumatic stress following an AE, comparative to levels reported in military personnel returning from conflict [4]. Similar findings have been reported in the Republic of Ireland (ROI), where surgeons describe high levels of anxiety, guilt, and low mood following an AE (*n* = 110).

Experiences of AEs are associated with increased burnout [6], leading to a higher likelihood of making subsequent errors [1] as well as moral injury, fear of reputational damage [6], and in some cases avoidance of certain procedures [7,8], or departure from the profession [8,9]. The impact of AEs on surgeons is likely compounded by the fact that surgeons do not readily access support in the aftermath [6]. Turner et al. [4] reported that 43% of surgeons sampled spoke to no-one following an AE. Limited engagement with support does not necessarily mean surgeons do not want to talk. A large survey of American paediatric surgeons revealed that 91% wished to be contacted after an AE, but 45% of them said no-one offered to speak to them [10].

Peer support programmes are reportedly the most desired form of support by healthcare workers following an AE [11]. Peer supporters may be uniquely positioned to understand the specific experiences of surgeons following an AE, having likely “been there” before [12]. Peer support programmes have been implemented successfully in broader healthcare settings [11,13,14] and successfully trialled in USA surgeon populations [12,15].

In light of this, The Royal College of Surgeons of England commissioned the first quality improvement (QI) collaborative, supporting participating hospitals to design, deliver, sustain, and evaluate, a peer support programme for surgeons after AEs. The SUrgeon Peer-led Post-incident Response Teams (SUPPORT) QI collaborative (January 2024 to March 2025), brought together surgical teams from 12 hospital Trusts/Groups across the UK and ROI. SUPPORT offered ‘delivered’ content as well as interactive opportunities for participants to share learning and reflect on challenges of implementation (see Table 1).

To our knowledge, SUPPORT is among the first UK and ROI based, multi-site, surgeon-specific peer support programmes of its kind. It aimed to support hospital Trust/Groups in setting up a pathway whereby surgeons affected by an AE were offered a one-to-one supportive conversation with a surgical peer. 3–6 participants from each hospital site (‘Site teams’) attended the SUPPORT events and were tasked with implementing the initiative within their hospitals. During the initiative, site teams trained peer supporters who offered a structured peer support conversation to any affected surgeon. No threshold of severity was set to trigger support, rather peer supporters were encouraged to recognise that the impact of an AE is often independent of severity and is determined by the significance attached to it by the affected surgeon. Peer supporters were trained to offer supportive listening, recognise psychological risk, signpost to appropriate pathways (such as in-house psychology provision) and offer a follow-up check-in conversation as needed.

To date, empirical evidence on the implementation and effectiveness of peer support for surgeons remains limited, particularly beyond two USA-based descriptive studies [12,15]. Qualitative research is notably lacking, despite its usefulness in understanding the ‘how’ and ‘why’ in implementation research [16]. The present study therefore explored site team members’ experiences of implementing a peer support programme for surgeons, specifically asking: (1) What are site team members’ perceptions of the usefulness and acceptability of the peer support programme? (2) What are the barriers and facilitators to the implementation of the peer support programme?

## 2. Materials and Methods

### 2.1. Participants

Eleven UK hospital Trusts and one ROI hospital group signed up to SUPPORT, with attendees from each Trust/Group (*n* = 55) forming site teams. Recruitment and completion of interviews took place between January and April 2025.

The recommended sample size for thematic analysis varies from 6–16 interviews [17] with a recent systematic review suggesting that saturation is usually reached by 9–17 interviews [18]. However, for interpretative types of qualitative analysis, sample size is often pragmatically shaped by resources and time [17]. Thus, all 55 potential participants were invited to participate, with sample size determined by those who consented to take part. The end sample (*n* = 16) was in line with the above recommendations, with no new distinctive patterns emerging in the data by the final interview.

Using purposive sampling, all site team members were contacted by e-mail and provided with study information. Site teams predominantly comprised surgeons, but also included clinical psychologists, anaesthetists and medical directors. Sixteen site team members consented and participated in an interview (9 female, 7 male). Most participants were surgeons (*n* = 13). The remaining participants were psychologists (n = 2) or anaesthetists (*n* = 1). Participants were from 10 of the 12 enrolled SUPPORT hospital sites (see Supplementary Material Section S1 for further participant demographics).

### 2.2. Procedure

This study received Bournemouth University ethical approval (Ethics ID: 60068). After obtaining informed consent, semi-structured interviews were held online using Microsoft Teams. An interview schedule was used as a template for discussions (see Supplementary Materials Section S2). This schedule was created by the lead author (GS) and refined by the research team to explore perceptions of usefulness and acceptability of SUPPORT (e.g., experiences of SUPPORT, aspects that were helpful/not helpful, differences between peer support offerings pre-post SUPPORT) and reflections on barriers and facilitators (what worked ‘well/less well’). Interviews were audio recorded, lasting between 39 and 82 min. Recordings were transcribed verbatim using a professional transcription service and anonymised for data analysis.

### 2.3. Data Analysis

Reflexive thematic analysis was used as a flexible approach for finding rich meaning in the data [19]. This provided a rigorous framework for data analysis, following six iterative steps: (1) Data familiarisation, (2) Generating initial codes, (3) Generating themes, (4) Reviewing potential themes, (5) Defining and naming themes, and (6) Producing the report [19]. Data analysis was carried out by GS, through prolonged engagement with the data [20]. GS kept a clear and transparent data trail of codes and identified themes/subthemes [21]. This was maintained principally with NVivo Pro 20 Release 1.6 software, but also through using comprehensive thematic tables to record key definitions, links and quotes for each theme/subtheme. To aid theme development, data analysis included second coding of one 1-h interview by MGH, and open discussion of codes to resolve any disagreements. Extensive discussion between GS and MGH occurred regularly throughout the analysis, to help with the development and refinement of themes/subthemes.

In terms of reflexivity, the lead researcher (GS) is a surgical trainee with an interest in surgeon wellbeing, and personal experience of an AE at work. GS is also a faculty member of the SUPPORT initiative. The analysis will have inevitably been conducted through the lens of a researcher with personal and professional experience of the subject matter, as well as familiarity with the site team members and their backgrounds. Interview conversations may have been influenced by the peer nature of discussions (i.e., participants discussing AEs with another surgeon), particularly given the power dynamic where the researcher was a more ‘junior’ colleague. Given GS’s background, a data driven approach to analysis was used, using guidance from frameworks for good practice during the data collection and coding [19,21,22,23]. GS’s prolonged engagement with potential participants during the first year of SUPPORT will have likely facilitated rapport and participant trust aiding credibility [21]. Member checking was also performed to help with confirmability; one participant read and commented on the results and outputs for this study.

## 3. Results

Four key themes were identified: Surgical Culture (Theme 1), The Importance of SUPPORT (Theme 2), Barriers (Theme 3), and Facilitators (Theme 4) to implementation. Each theme comprised subthemes, described below.

### 3.1. Theme 1: Surgical Culture

Participants considered how ‘surgical identity’ and stigma around perceived vulnerability influenced the implementation of the programme (see Table 2 for illustrative quotes).

#### 3.1.1. The Pressure of Professional Identity—“You’re a Rockstar at It, and Then You Move on (P2)”

The expectations of surgeons were discussed, particularly the deeply entrenched surgeon identity, to “ooze self-confidence” (P9), and show “bravado” (P6). This was reinforced by peers, colleagues, and culture. Professional identity was perceived to be threatened by any demonstration of vulnerability, particularly for leaders of surgical teams. Expectations of surgeons from others were also perceived to contribute to the pressure of appearing invulnerable. For example, one participant described how as a surgical leader they were not perceived to need support, as someone ‘who always copes’ (P11), further compounding the reliance on their ‘infallible’ character.

#### 3.1.2. The Shame and Stigma Around Support-Seeking—“There’s Something Wrong with Them for Being So Vulnerable…” (P12)

Some participants likened the perception of support-seeking to breaking an unwritten surgical rule to be “tough” (P12). Participants highlighted the stigma associated with support-seeking behaviour; there was a sense of shame and secrecy surrounding AEs, and reticence to admit mistakes to oneself and others. This served as a barrier to asking for help, with the emotional and psychological impact of accepting mistakes cited as particularly difficult. Despite this, some participants challenged these perceptions, suggesting that humility should be considered a desirable attribute in a surgeon; “you have to be brave to open up” (P8).

### 3.2. Theme 2: The Importance of SUPPORT

The existence of SUPPORT was perceived as necessary, valuable, and key in challenging stigma and normalising support-seeking (see Table 3 for illustrative quotes).

#### 3.2.1. Perceived Necessity of SUPPORT—“It’s of Great Value to the Profession” (P6)

There was a strong sense of the value of peer support, particularly in “talking to someone who gets it” (P4). Peer support was discussed as an essential part of surgical practice which has historically been lacking. This collective perception of SUPPORT’s necessity appeared to be grounded in several things, including the potential gravity of the impact of AEs. SUPPORT was seen as important for emotional recovery after AEs to maintain patient safety and effective team-working. The perceived justification for, and legitimacy of, SUPPORT was also frequently linked to the evidence base around surgeons not accessing support in the aftermath of AEs—something participants had not necessarily appreciated before their involvement in the project.

#### 3.2.2. The Impact of SUPPORT on Surgical Culture—“Actually, This Is How We Do Things Around Here” (P15)

Several participants noticed a shift in surgical culture which they attributed to the implementation of SUPPORT. Participants reflected on their changed views or behaviours since their involvement in SUPPORT. For example, one participant described feeling prompted to talk to a colleague in the immediate aftermath of a recent AE.

Participants also reflected on the existence of SUPPORT itself being a declaration of the need to prioritise surgeon wellbeing which, in turn, signalled a positive cultural shift. Given the widespread acknowledgement of the inevitability of AEs, several participants found SUPPORT instrumental in the normalisation of: (1) AEs occurring, and: (2) psychological reactions to AEs. The peer support model was also seen as valuable in challenging unhelpful attitudes towards support-seeking, in spreading the message that it is not “weak to ask for help” (P1).

### 3.3. Theme 3: Barriers to Implementation

Participants identified key barriers in implementing SUPPORT, including systemic lack of awareness of the need for support, difficulty identifying affected surgeons, and lack of time (see Table 4 for illustrative quotes).

#### 3.3.1. Systemic Lack of Awareness and Institutional Integration—“Really? Surgeons Need Help? Support? Really? Why?” (P6)

There was a general lack of institutional awareness of the importance of supporting surgeons after AEs and of SUPPORT. The gap between intention and integration, both systematically and culturally, was illustrated by one participant not being approached by their SUPPORT colleagues after an AE during the live phase of the programme: “The other two didn’t even ring me to ask me how I was…” (P11). This suggests that supportive practice and behaviour was not yet enmeshed between directly involved parties. This may reflect the barrier of cultural norms around senior colleagues’ abilities to cope without needing support (subtheme 1.1) as well as the logistical challenge of time pressures of delivering peer support (subtheme 3.2). It also highlights the potential impact of surgeon seniority and experience in influencing assumptions around the need for support.

Garnering institutional support for SUPPORT was similarly difficult for some participants, where the importance of supporting surgeons was not prioritised. However, institutional backing for SUPPORT and financial investment helped to prioritise the “need to show that we care for our doctors…” (P8).

#### 3.3.2. Practical and Logistical Challenges—“I Think I Can Spend More Time on This, but I Don’t Have Time” (P11)

The lack of time available to site teams was unanimously identified as a prominent barrier to the successful implementation of SUPPORT. There was a sense that SUPPORT “never quite got to the top of anyone’s to-do list….” (P16) and suffered through lack of protected time. The difficulty of scheduling peer support training was also consistently articulated by participants, as well as the practical challenge of staff turnover. The latter directly affected the cohort of peer supporters and threatened the sustainability of the programme.

#### 3.3.3. Peer Support Training—“…We’re All a Bit Scared of Saying the Wrong Thing?” (P13)

Lack of confidence in delivering peer support training was perceived as a challenge to delivery of SUPPORT, particularly when delivering a peer support conversation, where participants feared “saying … or doing the wrong thing” (P13). While participants expressed concern about SUPPORT feeling like a ‘tickbox’ exercise, there was perceived value in the resources provided for peer support training, including the structured checklist for conversations.

#### 3.3.4. Difficulty in Referral to SUPPORT—“We’re Missing Access to Surgeons Who Really Need Us, Somehow” (P2)

Several participants highlighted the challenges in identifying both AEs and affected surgeons. Participants were not aware of a robust mechanism of AE capture which would give certainty that surgeons potentially needing support could be identified. There were differing views about appropriate referral to SUPPORT. Some participants took a uniform, opt-in approach, but highlighted the issue of missing important contact with surgeons in need of SUPPORT. Some site teams set up dedicated e-mail addresses for opt-in, while also proactively contacting affected surgeons if they were informed by word-of-mouth. Others pointed out the pros and cons of an opt-out mechanism in terms of a mandatory, yet proactive approach. While many site team members described the difficulty of identifying surgeons following an AE, some hospital Trusts/Groups used strategies beyond word-of-mouth for SUPPORT referral, such as identifying surgeons via division quality meetings, mortality and morbidity meetings, through involvement of Clinical/Medical Directors in the SUPPORT faculty, or via referral routes from a key person in theatre (such as Clinical Nurse Manager).

### 3.4. Theme 4: Facilitators to Implementation

Key facilitators were identified by participants in implementing SUPPORT, including the influence of senior site team members for institutional endorsement, practical infrastructure and collaboration with psychology colleagues (see quotes in Table 5).

#### 3.4.1. Influence of Site Teams—When Championing SUPPORT “It’s Important to Have a Role of Responsibility.” (P3)

The critical role of well-connected, often senior, site team members, was recognised, particularly in engaging institutional backing for SUPPORT and actively driving the implementation of the programme. Several participants had worked in their organisations for many years and felt this was beneficial in introducing the initiative and having influence within their hospital. Having site team members with leadership/managerial roles also helped in identification of AEs.

#### 3.4.2. Support Structures: Administrative, Psychology and Royal College Backing

Administrative support was described as a key practical facilitator, in promoting the project, co-ordinating referrals, and contacting relevant peer supporters. The fact that RCS England led the SUPPORT initiative was also seen as an advantage. The importance of this was not only in the structure the programme afforded but in its credibility; having “the stamp of the Royal College on it is a very good start” (P10). This was felt to be linked to external perceptions of the programme’s value.

Support from, and collaboration with, psychology colleagues was seen as key to successful implementation to SUPPORT in organisations where this was possible. This added credibility to the programme in having “some science behind it” (P13). Several participants identified the benefits of psychologists’ unique insights about peer support delivery, and their crucial role in supporting the peer supporters, particularly given the psychological impact that providing support could sometimes have. Psychology input for SUPPORT was therefore recommended, particularly following positive experience of accessing psychology support and the belief that psychology input may boost the confidence of peer supporters.

### 3.5. Model and Synthesis of Findings

**Figure 1:**
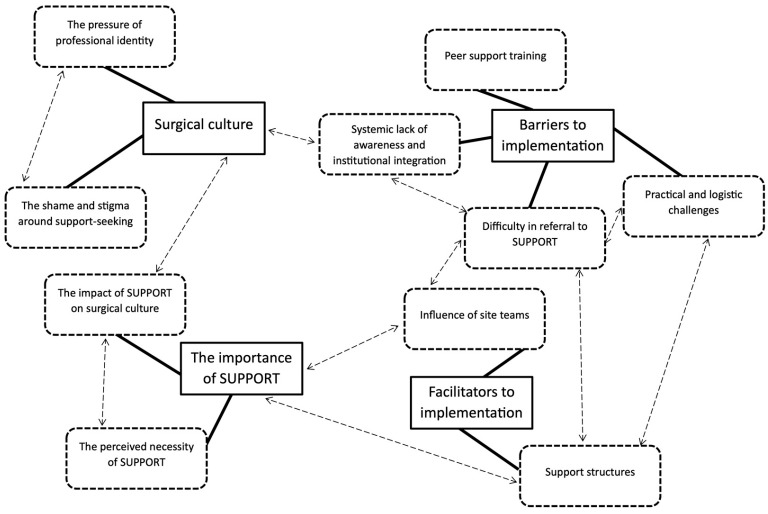
Thematic Map of Themes and Subthemes.

Figure 1 provides a synthesis of the key themes and subthemes from these findings. Surgical culture, encompassing stigma surrounding help-seeking and pressure of “professional” identity, was perceived to lead to a systemic lack of awareness and institutional integration of SUPPORT which in turn reinforced the non-supportive culture. Together, these factors were seen as key barriers to identifying affected surgeons and to effective implementation. Lack of confidence in delivering peer support, alongside logistical challenges such as insufficient protected time, were also perceived to hinder referral and uptake. Conversely, existing support structures were identified as important facilitators of implementation. These included administrative support, endorsement from the RCS, and integration with psychology colleagues. These structures had potential to mitigate logistical challenges (for example, administrative support offsetting time constraints) and were also seen to enhance the perceived legitimacy and necessity of SUPPORT (e.g., with psychology adding ‘science’ behind the initiative).

The involvement of well-connected, often senior, site team members was also highlighted as a facilitator to implementation. These individuals were perceived to play an important role in signalling the importance of support and promoting cultural change within surgery. Senior team members also helped identification and referral of affected surgeons through their positions within formal meeting structures. Despite the challenges to implementation, SUPPORT was seen as necessary for providing important support for surgeons. Perhaps vitally, SUPPORT was perceived as both challenged by, and an important driver of, change to surgical culture.

## 4. Discussion

This study explored surgical site team members’ perceptions of the usefulness and acceptability of a peer support programme following AEs, identifying perceived barriers and facilitators to its implementation. The findings revealed a strong collective belief in the importance of SUPPORT but highlighted a perceived disconnect between conceptual endorsement and the practical, institutional, and cultural challenges of implementation.

### 4.1. Surgical Culture and the Need for SUPPORT

Consistent with previous research [1,6,15] surgical culture emerged as a key barrier to support-seeking. While participants unanimously endorsed SUPPORT, surgical identity impeded surgeons’ abilities to seek support. Surgeons were seen to be ‘tough,’ with support-seeking seen as a sign of ‘weakness.’ While norms of stoicism and infallibility have been reported elsewhere [24], our findings describe the tension between a perceived culture that reinforces silence and avoidance after AEs, and the growing recognition that recovery after an AE is crucial for patient safety and effective team-working. Thus, whilst previous work has highlighted the lack of support-seeking in surgeons [1], our study helps to understand why this may be.

A key perceived benefit of SUPPORT was its role in normalising AEs, surgeons’ responses to them, and subsequent support-seeking. This aligns with previous work, emphasising the value of validating affected surgeons’ experiences [15] and the role of talking to someone who has “been there” [12]. Indeed, SUPPORT was seen as more than a reactive tool, serving as a mechanism for cultural change—as a powerful statement against blame culture and stigma.

### 4.2. Barriers and Facilitators to Implementation

Participants described a clear mismatch between the perceived necessity of SUPPORT and the persistent barriers to its operational uptake. Lack of institutional support was described as a clear barrier, seemingly related to limited awareness of the need to support surgeons. Like other QI collaboratives [25] and peer support interventions [26], the need for protected time and administrative support were viewed as key to success. These barriers align with the COM-B model of behaviour change [27,28]; participants reported capability (knowledge), and motivation (willingness to support), but had limited opportunity (due to resources, time, and systemic lack of awareness).

Integration with psychology colleagues, the influence of site teams and institutional support were also identified as important facilitators. Consistent with previous work [29] senior leadership backing enhanced the legitimacy, visibility and traction of SUPPORT. This suggests strategic alignment with executive leadership, including framing SUPPORT as a workforce retention and risk management strategy, may be integral to sustainability.

Participants also highlighted difficulties in initiating appropriate support, particularly regarding identifying affected surgeons. While some participants perceived overly formalised opt-out systems as intrusive, other participants felt strongly that surgeons should be contacted after an AE. Indeed, placing the onus on an affected surgeon to access support in the aftermath of an AE has been identified as a potential barrier elsewhere [1], with recommendations for an active “push” rather than “pull” strategy [30]. This aligns with participant accounts emphasising the need for proactive contact with affected surgeons.

### 4.3. Mapping Findings to Existing Implementation Research

Our findings map closely onto key domains of the Consolidated Framework for Implementation Research (CFIR, ref. [31]). In relation to intervention characteristics, a perceived strength of SUPPORT was its capacity to challenge surgical culture. Participants described SUPPORT as a mechanism for normalising responses to adverse events and support-seeking. However, concerns were raised about confidence in delivering peer support conversations. This reflects a recognised barrier in the literature, where healthcare professionals may feel insufficiently equipped to deliver interventions [32,33]. While uncertainties remained regarding optimal implementation processes (such as identifying affected surgeons), perceptions of the usefulness of SUPPORT were shaped primarily by factors within the inner setting and individual characteristics.

Within the inner setting, strong management and institutional support were perceived to enhance the visibility and legitimacy of SUPPORT, consistent with broader hospital-based interventions [33,34]. Conversely, surgical culture (particularly norms about infallibility and stigma around support-seeking) emerged as key barriers to implementation. At the individual level, staff commitment and belief in the initiative were key facilitators, consistent with previous research [35,36]. While professional identity was seen to hinder support-seeking, there was widespread recognition of the value of SUPPORT. Taken together, these findings suggest that successful implementation was perceived to be shaped by organisational resources, leadership alignment, and perceived competence in delivering peer support, as well as by deliberate efforts to shift cultural norms and engagement practices.

### 4.4. Implementation Recommendations

This study suggests that successful implementation of surgeon-specific peer support interventions may benefit from targeted site-level actions including provision of protected time for site teams, dedicated administrative support, senior leadership involvement, and access to comprehensive training for peer supporters. Collaboration with psychology was also viewed as important, both in strengthening the scientific credibility of SUPPORT and in supporting its delivery.

At a broader level, institutional backing was perceived as critical for the implementation of SUPPORT. Identifying surgeons affected by an AE was also universally highlighted as a key implementational challenge. Several potential referral pathways may help to address this, such identification by key operating theatre team members, at procedural meetings (i.e., mortality and morbidity meetings), and via formal investigative processes. Participants had mixed views on opt-in versus opt-out referral for peer support, reflecting the tension between promoting engagement while avoiding intrusiveness or confidentiality concerns. These challenges may be mitigated by ensuring that the content of SUPPORT conversations is not documented, setting clear confidentiality boundaries before SUPPORT conversations, and using clear opt-out language when SUPPORT meetings are offered. Collectively, these strategies may support more systematic, accepted, and proactive engagement with surgeons following AEs.

### 4.5. Strengths, Limitations and Future Research

This study highlights the need for interventions that address cultural barriers to support seeking for surgeons following AEs. It is, to our knowledge, the first in-depth qualitative study of exploring implementation of a surgeon-specific peer support model following AEs in the UK and ROI. However, several limitations should be considered. This study reflects the perspectives of surgical site team leads, rather than surgeons receiving the support. Selection bias should also be noted, as participants enrolling in this study were likely to be more engaged within the SUPPORT initiative. As a result, interviews may overrepresent more positive experiences of SUPPORT. There may also be social desirability bias, given the researchers were part of the SUPPORT faculty.

Although this was a multi-site initiative, the analysis did not explicitly examine site level differences in the setup of SUPPORT. Quantitative data on this aspect, and its impact for SUPPORT implementation, would also be useful. Future work is needed to explore the experiences of individuals who are less engaged with SUPPORT, as well as supported surgeons whose voices are integral in understanding the true value of the initiative. Researchers would benefit from hearing from surgeons who did not access support, even when it was available, to better understand perceived barriers to uptake. Additionally, research is needed to understand the impact of SUPPORT on the wider surgical team and ultimately on patient care.

Finally, more data are needed regarding the effectiveness of peer support programmes in driving change, not only for individual surgeons, but more broadly for surgical culture. While there is some promising data from small scale USA studies (e.g., ref. [12]), UK-based evidence remains limited. Future mixed methods research should focus on: (1) the perceived usefulness of peer support conversations from recipients, (2) patterns of uptake, (3) quantitative contextual drivers affecting engagement, and: (4) longitudinal measures of organisational change in attitudes towards support seeking. Such data will be important in understanding the impact of peer support on individual surgeons and organisational culture.

## 5. Conclusions

This study provided novel insights into implementing a multi-site peer support initiative for surgeons after AEs. SUPPORT was seen as a powerful statement against the culture of stigma and blame, by normalising surgeons’ responses to AEs and encouraging support-seeking. While institutional support and protected time were identified as facilitators to implementation of SUPPORT, the perceived need for improved systems to identify surgeons affected by AEs was also evident. These findings underscore the perceived importance of interventions that address both structural and cultural barriers, promoting acceptance of support-seeking as a routine and constructive response to adverse events.

## Figures and Tables

**Table 1:** Outline of SUPPORT’s content and resources.

Session	Topics Covered ^1^
Pre-launch Event	Introduction to the aims of the collaborative and the psychology behind SUPPORT.
In-person Launch Day	Overview of the SUPPORT Collaborative Introduction to evaluation of SUPPORT Overview of the SUPPORT peer conversation Understanding normal reactions to AEs Introduction to managing psychological risk in difficult conversations
Online Webinar Half-day 1	The SUPPORT peer conversation and checklist Supporting peer supporters Collaborative Milestones SUPPORT evaluation
In-person Day 2	Importance of SUPPORT Simulating SUPPORT conversations and role-plays Confidentiality and SUPPORT Implementing organisational change
Online Webinar Half-day 2	Medico-legal considerations Diversity in SUPPORT Sustaining SUPPORT
In-person Day 3	The Psychology of high performing teams Supporting colleagues attending inquests Views on failure and forgiveness Common challenges in SUPPORT
In-person Celebration Event	Making Sense of Mistakes SUPPORT Evaluation data Patient Safety Incident Response Framework The second victim terminology Planning SUPPORT 2

^1^ The programme included group discussion, sharing personal stories, presentations, role-play activities, and guest speaker sessions. SUPPORT attendees were also provided with online small group support sessions, eight online PowerPoint Modules, videos, leaflets and a conversation checklist to support the implementation of SUPPORT. All sessions outlined in Table 1 were core SUPPORT sessions, with online small group sessions included as an additional optional resource.

**Table 2:** Theme 1: Surgical Culture-illustrative quotations.

Subtheme	Quotation
1.1 The pressure of professional identity	“It’s a bravado thing. I see it so often. I think there is this stiff upper lip attitude in surgery of, ‘We must carry on. Just buck up. Carry on.’” (P6)
	“You’ve got to ooze self-confidence for people to believe in you, and that doesn’t come then very well with humility and vulnerability of, “I’ve f***ed up and it’s all gone horribly wrong.”” (P9)
	“You’re a rockstar at it, and then you move on. You don’t get too emotionally involved, in order to succeed.” (P2)
	“[Following an AE] … the response I had from one of the colleagues was, ‘But you’re P11, you always cope with everything.’ You know? And I kind of felt, that’s actually putting more pressure [on me], that’s not helpful, is it?” (P11)
1.2 The shame and stigma around support-seeking	“The hidden curriculum is you have to be tough, you shouldn’t need these kinds of supports and there’s something wrong with you if you do.” (P12)
	“It’s like [surgeons] have to carry on, have this suit of armour, and not be affected. If they are, then there’s something wrong with them for being so vulnerable and maybe they should go off and do psychiatry or something.” (P12)
	“I think fear is huge. I think shame is another thing. I think loss of confidence and having to admit your failures or that you’re human and that can be very difficult.” (P15)
	“…definitely, some people I know, maybe it’s our older generation, or previous one, they think it’s a sign of weakness, which it’s not actually, you have to be brave to open up.” (P8)

**Table 3:** Theme 2: Importance of SUPPORT-illustrative quotations.

Subtheme	Quotation
2.1 The perceived necessity of SUPPORT	“…we know we want to prevent suicides, that is the real end result of some of these AEs… it’s not a fantasy. It actually happens. But… below that, there are people completely broken who never get back to the level that they were at… it is very, very serious.” (P9)
	“I think it’s been hugely under-recognised, under-reported and I think it’s of great value to the profession.” (P6)
	“There is certainly a sense that you’re talking to somebody who gets it and who knows what it’s like in the trenches day-to-day and who knows the kind of responsibility you have and how it is very different when you operate on someone (P4)
	“This is why this is important. It’s about keeping people at work, keeping them productive, looking after our patients properly, and creating the right environment for the Trust.” (P6)
	“…having been told more about the available literature, I now appreciate why surgeons need this bit of special additional help… to try and improve their journey back to normal function.” (P1)
2.2 The impact of SUPPORT on surgical culture	“There’s a much better recognition now, because of this. A lot of people now seem to know about it and understand its importance.” (P6)
	“[my involvement in SUPPORT] …dispelled some of my stereotypical ideas about what surgeons are and what they’re not.” (P2)
	“I think it will allow everyone to see that it is not weak to ask for help, it is not weak.” (P1)
	“Actually, this is how we do things around here” (P15)
	“…this makes it that this is acceptable, and this is normal. Things can go wrong at times, and when it goes wrong, when you are in difficulty, there is help available.” (P8)

**Table 4:** Theme 3: Barriers to Implementation- illustrative quotations.

Subtheme	Quotation
3.1 Systemic lack of awareness and institutional integration	“…a practical issue we find is just trying to make sure people know about [SUPPORT]. We still haven’t completely resolved that.” (P13)
	“They see things very much as bums on seats… until the impact is felt, and somebody is actually off long-term sick because of the impact of it, I don’t think they’d really consider the psychological needs of the surgeons at all.” (P16)
	“Then I had one [an elective death] and…the other two didn’t even ring me to ask me how I was…” (P11)
	“When I first raised it at senior level, there were eyebrows of, ‘Really? Surgeons need help? Support? Really? Why?’” (P6)
	“Not everybody would be willing to do that [secure funding for SUPPORT]. But the medical director felt that this is something, that we need to show that we care for our doctors as well.” (P8)
3.2 Practical and logistical challenges	“[I feel] …guilt, about the fact that that money has come in, it was my idea to join in, and yet we haven’t really properly launched it yet.” (P9)
	“It never quite got to the top of anyone’s to-do list list just because of the huge pressure we’re under with everything else going on.” (P16)
	“There needs to be …negotiations within Trusts and within departments about the time that’s required and recognising that time in some way.” (P4)
3.3 Peer support training	“Just getting all the interested parties in the same place at the same time for as long as it takes [for training] was quite challenging” (P6)
	“…people are quite nervous about having that conversation …we’re all a bit scared of saying the wrong thing or doing the wrong thing” (P13)
	“Feeling that we were adequately trained to provide the training to our colleagues, we were worried about.” (P16) “We’re not trained counsellors so a lot of that was using the College resource which you all provided, which actually is really good.” (P4)
3.4 Difficulty in referral to SUPPORT	“The bit that’s still really difficult that we haven’t nailed is how to make sure that we get people who have had an incident, definitely offered the opportunity to use SUPPORT.” (P13)
	“I think the main thing…is feeling like we’re missing access to surgeons who really need us, somehow” (P2)
	“When it comes to the self-referral bit, you know, how many people are actually going to write an email saying, “I need help with this”? That’s just probably not going to happen very often at all.” (P4) “[SUPPORT is not] …the sort of thing you can force on people. I’d hate to see it be a formal process where, if something goes wrong, you must be referred to it.” (P6) “[SUPPORT should be offered] …automatically without the surgeon involved having to instigate it….” (P15)

**Table 5:** Theme 4: Facilitators to Implementation- illustrative quotations.

Subtheme	Quotation
4.1 Influence of site team members	“…sharing this work with a very senior surgeon… has been really positive, because of their influence and connections.” (P2) “Dare I say it, I think I had a bit of influence. Because I’m now 20 years at the Trust…. I think, generally, people know me…. And they know if they help me, I’ll help them.” (P6)
	“It’s important to have a role of responsibility” (P3)
	“…[X] is an associate medical director who sits on the SIU panel and various other committees. So, they actually do get to hear about difficult situations that have occurred in the Trust.” (P9)
4.2 Support structures	“[with admin support] …trying to set up meetings, arranging sending out leaflets, making posters, setting up an e-mail has been incredibly easy.” (P7)
	“[the psychologist] viewpoint is to bring something different… it has some science behind it” (P13)
	“I think having it led by the College has made a big difference to the impact, both from the surgeons’ perspective, but also the Trust’s perspective.” (P4)
	“We think that as much as peer-to-peer surgeon support is useful, it needs to be backed up with some more formal psychological support as well.” (P4)

## Data Availability

To protect participants’ privacy for interview data, full datasets are not uploaded to a database. Please contact the corresponding author for access to anonymised data excerpts and thematic coding tables for use in secondary analyses or data triangulation.

## Supplementary Materials

The following supporting information can be downloaded at: https://www.mdpi.com/article/10.3390/healthcare14091220/s1, Supplementary Materials S1: Demographic information; Table S1: Further demographic information for participants; Supplementary Materials S2: Semi-structured interview schedule.

## Abbreviations

The following abbreviations are used in this manuscript:

SUPPORT	SUrgeon Peer-led Post-incident Response Teams
QI	Quality Improvement
AE	Adverse event
RCS	Royal College of Surgeons
UK	United Kingdom
ROI	Republic of Ireland
USA	United Stated of America
