# Supplementary material for: “Talking to Someone Who Gets It…” Experiences of Surgical Site Teams in Implementing a Peer Support Programme for Surgeons After Adverse Events
Source: Healthcare (Basel). 2026 May 1;14(9):1220. doi: 10.3390/healthcare14091220 (PMC13164216; doi:10.3390/healthcare14091220)
Supplement: Supplementary file 1 [file healthcare-14-01220-s001.zip › healthcare-4190190-supplementary.pdf]

Supplementary Materials S1: Demographic information.

Table S1: Further demographic information for participants.

Profession	Specialty	Gender	Hospital Site
Consultant Surgeon	Head and Neck	Female	1
Psychologist	Staff Wellbeing	Female	2
Consultant Surgeon	Urology	Female	2
Consultant Anaesthetist	Anaesthetics	Female	3
Consultant Surgeon	Vascular	Male	3
Consultant Surgeon	Trauma and Orthopaedic	Male	3
Consultant Surgeon	Urology	Male	4
Specialist Registrar	Urology	Female	5
Consultant Clinical Psychologist	Staff Wellbeing	Female	5
Consultant Surgeon	Oncoplastic Breast	Male	6
Consultant Surgeon	Urology	Male	6
Consultant Surgeon	Urology	Male	7
Consultant Surgeon	Head and Neck	Female	7
Consultant Surgeon	Maxillofacial	Male	8
Consultant Surgeon	Breast	Female	9
Consultant Surgeon	Urology	Female	10

Note: to protect participant anonymity for quotes, the demographic information has not been linked to, or ordered by, participant number. Hospital sites have been presented by pseudonym number rather than site name.

9 participants also described holding a current leadership role within their hospital; 5 as clinical leads for their specialty/area, 2 as programme directors for surgical training, 1 as director for a hospital mandatory training programme (human factors in patient safety) and 1 as the deputy medical director.

Supplementary Materials S2: Semi-structured interview schedule

Note: this is an indicative guide of the types of topics that will be covered and questions that will be asked in the semi-structured interviews.

Opening

Thank you for participating in this research. The purpose of this project is to evaluate the Surgeon Peer-led Post-incident Response Teams (SUPPORT) Improvement Collaborative, launched by RCS England in January 2024 in collaboration with Bournemouth University (BU). As part of our evaluation, we are carrying out interviews with members of the SUPPORT project team at participating sites. These interviews will give us a valuable opportunity to learn about your experience of and honest feedback about being involved with SUPPORT.

Your participation is entirely voluntary, and you can withdraw from the interview at any time. You do not have to answer any question or questions you don't wish to answer. You have already signed an online consent form, but before we begin, can I confirm you are still happy to take part in the interview?

This interview will be recorded. The audio recordings will be stored on a password-protected computer and will only be accessible to the research team and professional transcription service. Recordings will be deleted as soon as they have been transcribed. Our data will be analysed by collating all interview responses and identifying broad recurring themes. You will not be able to be identified in any external reports or publications about the research.

Before we start, is there anything you would like to ask or clarify? I will begin the audio recording now.

Interview

Could you tell me a bit about your current role? For example, how long have you been doing it?

What are your thoughts about the importance of supporting surgeons after adverse events?
What do you think supporting surgeons after adverse events should look like?
What are your thoughts about what it should look like for trainees?
What do you think the barriers to accessing support are?
Follow-up: what about Could you tell me about what made you want to be involved with the SUPPORT project?
for trainees?

What do you think of surgeon-led peer support as a model?
Follow-up: could you tell me about alternative support models that could be effective?

Could you tell me about what made you want to be involved with the SUPPORT project?
Before you signed up to the SUPPORT Improvement Collaborative, what did supporting surgeons after adverse events look like in your Trust?
Follow-up if Trust had existing support mechanism e.g. what has been tried in the past, was this effective?
Could you tell me a bit about how your organisation prioritises support for surgeons after adverse events?
Follow-up if not the case e.g. could you tell me a bit more about that?

Can you tell me about SUPPORT in your organisation?
How do you find out about adverse events?
How are surgeons referred?
How would you describe your role in the SUPPORT project?
Who else is involved?

How would you describe the implementation of SUPPORT in your organisation?
Could you tell me about things that have gone well?
What do you think has facilitated this?
What have the challenges been?
Follow-up questions/prompts depending on responses to do with different stages:
What has worked well/less well in terms of creating a SUPPORT team?
Advertising/publicising SUPPORT?
Training peer supporters?
Initiating/uptake of SUPPORT conversations?
Facilitators/barriers to changing uptake/culture
Evaluation?
Sustainability?

How would you describe the support available to surgeons after adverse events in your organisation now?
Can you tell me about the experience of being part of the SUPPORT collaborative?
What aspects of being involved in the collaborative have been useful?
What aspects have not been useful?
What would you change about the SUPPORT project?
For the final question, what could we improve for SUPPORT 2?

Is there anything else that we haven't touched on that you would like to talk about?
Do you have any questions?

Closing

Thank you very much for giving up your time to participate in this research. We will be sending you a debrief document by email, but please do get in touch if you have any further questions or concerns. Unless you have any questions at this point or there is anything else you would like to discuss, I will stop recording now.
